# Effects of Occupational Noise Exposure on 24-Hour Ambulatory Vascular Properties in Male Workers

**DOI:** 10.1289/ehp.10346

**Published:** 2007-07-23

**Authors:** Ta-Yuan Chang, Ta-Chen Su, Shou-Yu Lin, Ruei-Man Jain, Chang-Chuan Chan

**Affiliations:** 1 Department of Occupational Safety and Health, College of Public Health, China Medical University, Taichung, Taiwan; 2 Department of Internal Medicine, National Taiwan University Hospital, Taipei, Taiwan; 3 Institute of Environmental Health, College of Public Health, China Medical University, Taichung, Taiwan; 4 Institute of Occupational Medicine and Industrial Hygiene, College of Public Health, National Taiwan University, Taipei, Taiwan

**Keywords:** ambulatory arterial stiffness, ambulatory vascular properties, automobile workers, occupational noise exposure, panel study

## Abstract

**Background:**

Epidemiologic studies have demonstrated that occupational noise exposure is associated with hypertension, but the related mechanism in vascular structural changes is unclear.

**Objective:**

This panel study aimed to investigate effects of occupational noise exposure on ambulatory vascular structural properties in male workers.

**Methods:**

We recruited 20 volunteers and divided them into a high-noise–exposure group of 15 and a low-noise–exposure group of 5 based on environmental noise measurement in an automobile manufacturing company. We determined individual noise exposure and measured personal ambulatory vascular property parameters simultaneously during 24 hr. Linear mixed-effects regression models were used to estimate transient and sustained effects of noise exposure on vascular parameters by adjusting some confounders collected from self-administrated questionnaires and health checkups.

**Results:**

The high-noise–exposed (85 ± 8 dBA) workers had significantly higher systemic vascular resistance (SVR) than the low-noise–exposed workers (59 ± 4 dBA) during work and sleep periods. Contrarily, low-noise–exposed workers had significantly higher brachial artery compliance (BAC), brachial artery distensibility (BAD), and systemic vascular compliance (SVC; marginal, *p* = 0.07) than high-noise–exposed workers during off-duty periods. We also found that high-noise–exposed workers had significantly lower BAC (1.38 ± 0.55 %mL/mmHg) and BAD (1.29 ± 0.51 %/mmHg), as well as lower SVC (0.24 ± 0.10 mL/L/mmHg), but higher SVR (1.93 ± 0.67 mL/L/min) compared with low-noise–exposed workers over a 24-hr period.

**Conclusions:**

Our findings suggest that in automobile workers, occupational noise exposure may have sustained, not transient, effects on vascular properties and also enhances the development of hypertension.

Several epidemiologic studies have reported that exposure to noise is associated with cardiovascular disease, including myocardial infarction and coronary heart disease (Babisch et al. 2005; Davies et al. 2005; Virkkunen et al. 2005; Willich et al. 2006). This association may be because noise exposure enhances the development of hypertension. Many field studies have demonstrated that high-level noise exposure [> 85 A-weighted decibels (dBA)] may cause the transient elevation of blood pressure (Fogari et al. 2001; Green et al. 1991; Lusk et al. 2004). More industry-based epidemiologic studies suggest that noise exposure causes sustained elevation of workers’ blood pressure (Fouriad et al. 1984; Jonsson and Hansson 1977; Lusk et al. 2002; Talbott et al. 1999; Tomei et al. 2000; Verbeek et al. 1987; Wu et al. 1987; Zhao et al. 1991). In a previous study (Chang et al. 2003), we found transient and sustained increases of systolic blood pressure (SBP) after occupational noise exposure at > 85 dBA among automobile workers.

One possible biological mechanism of hypertension caused by noise exposure is sympatheticotonia-induced endothelial lesion. Acute noise exposure activates a sympathetic reflex immediately (Andrén et al.1980; Baudrie et al. 1997; Casto et al. 1989), accelerates the development of structural changes in the cardiovascular system, and then induces a sustained elevation of blood pressure (Andrén et al. 1980; Baudrie et al. 1997; Jonsson and Hansson 1977).

However, the structural changes in vascular properties related to sympatheticotonia-induced mechanisms of hypertension are not clear. Because macro- and microvascular changes in arterial function and vascular physical properties caused by hypertension occur before the development of clinical disease (Berenson et al. 1992), new approaches have been developed to measure vascular stiffness, such as arterial compliance and distensibility (Riley et al. 1986; Wada et al. 1994). It is of interest to study the early changes in vascular properties because reduced arterial compliance and distensibility leads to increased SBP, left ventricular hypertrophy, and acceleration of arteriosclerosis (Urbina et al. 2002, 2005).

Some experimental studies have reported the significant increase of total peripheral vascular resistance after noise exposure among normotensive males (Andrén et al. 1980; Sawada 1993a, 1993b), but their results were limited to an intermittent (10–20 min) exposure to a dose of > 95 dBA of noise on resting vascular properties. The purpose of the present study was to build on previous findings and investigate the effects of occupational noise exposure on ambulatory vascular structural properties and to elucidate the possible mechanism of hypertension in automobile manufacturing workers.

## Materials and Methods

### Subjects

The recruitment and selection of workers from an automobile company as study subjects have been described previously (Chang et al. 2003). In short, an industry-based occupational hygiene investigation determined that the noise levels were at 79–110 dBA and that ototoxic chemicals were not present in the work environment of this automobile company. Twenty male workers were randomly selected from all male employees, including 15 subjects with high-noise exposure from operational units performing press forging, engine manufacturing, assembly, body assembly, and roller and track trial testing; 5 subjects with low-noise exposure were selected from the staff working in a separate office building. Because our monitoring protocol required all participants to carry their noise dosimeters and ambulatory vascular property monitoring devices simultaneously on and off work for 24 hr, we had to limit the number of our study subjects in order to ensure high compliance.

According to health check-up results in 2000 before conducting this study, none of these 20 subjects was diagnosed with hypertension or other cardiovascular diseases. The check-up also provided important information related to hypertension, such as each subject’s height and weight, resting blood pressure, total cholesterol, and triglyceride. Individuals’ heights and weights were used to calculate body mass index (BMI). For subjects working in sites with environmental noise levels > 85 dBA, audiometric tests were performed at 500, 1,000, 2,000, 3,000, 4,000, 5,000, and 6,000 Hz for both ears annually. During the panel study, we used a questionnaire to collect additional risk factors of blood pressure, such as age, employment duration, exercise habits, smoking history, alcohol consumption, and family disease history. The Institutional Review Board of the College of Public Health, National Taiwan University, approved this study, and written informed consent was obtained from each participating worker.

### Ambulatory vascular property monitoring and recording

We recorded the readings of each subject’s vascular parameters related to structural properties every 30 min during waking time (0800–2300 hours) and every 60 min during sleeping time (2300–0800 hours) repeatedly recorded using a portable, non-invasive, automated monitoring and recording system (DynaPulse model 5000A; Pulse Metric, San Diego, CA, USA). The vascular parameters included brachial artery compliance (BAC; %mL/mmHg), brachial artery distensibility (BAD; %/mmHg), brachial artery resistance (BAR; mmHg/L/min), systemic vascular compliance (SVC; mL/L/mmHg), and systemic vascular resistance (SVR; mL/L/min). Arterial compliance and distensibility are associated with vascular stiffness, left ventricular hypertrophy, and arteriosclerosis (Urbina et al. 2002, 2005). The DynaPulse system can measure a subject’s arterial pulsation signals, known as the arterial waveform, through a noninvasive cuff device. The curve data for automated offline analyses of brachial artery pressure were performed to calculate brachial artery distensibility (Urbina et al. 2002, 2005).The pressure waveform was then calibrated and incorporated into a physical model of the cardiovascular system that has been validated against separate data collected in a cardiac catheterization laboratory (Brinton et al. 1997), assuming the straight tube brachial artery and T-tube aortic system. Previous validation studies of the DynaPulse instrument demonstrated high correlation between compliance (from which distensibility was calculated) measured with cardiac catheterization and those derived by noninvasive means (*r* = 0.83) (Brinton et al. 1997, 1998). The intraclass correlation coefficient for blind duplicate recordings was 0.72, indicating that most of the variability in measurement was due to inter-individual variation (Urbina et al. 2002, 2005). Each study subject carried a Dynapulse system for 24 hr to complete the continuous monitoring of structural changes in vascular properties. We used the vascular parameter data of 15 high-noise–exposed subjects during the working period (0800–1630 hours) to investigate the transient effect of occupational noise exposure on workers’ vascular properties. We used all of the participants’ vascular parameter measurements over 24 hr to study the sustained effect by occupational noise exposure.

### Exposure measurements

We measured personal noise exposure continuously using a personal noise dosimeter (Logging Noise Dose Meter Type 4443; Brüel & Kjær, Nærum, Denmark), which can report 5-min continuous equivalent sound levels (Leq) at an exchange rate of 3 dBA and the time-weighted averages (TWAs) of noise doses. All subjects carried the Logging Noise Dose Meter to measure their personal noise exposure with 5-min readings over their working periods (0800–1630 hours). Due to limited data-logging memories, one single TWA noise exposure level was set up for each subject during off-duty periods (1630–2300 hours). We presumed nondifferential noise exposure in both groups and did not measure noise exposure levels during sleep periods. To investigate the time-lag effect of acute noise exposure, 5-min exposure measurements were summarized into 30-min and 60-min time-moving-average segments for further analysis.

### Statistical analysis

We performed univariate comparisons between the two exposure groups using *t*-tests for continuous variables and Fisher’s exact test for dichotomous variables. We used linear mixed-effects regression models to associate transient noise exposure with vascular parameters by controlling important confounding factors of study subjects (Littell et al. 1996). The linear mixed-effects regression model has the advantages of dealing with the autocorrelation problem between repeated vascular parameter measurements and increasing statistical power by combining information across study subjects. In our mixed-effects models, personal noise exposure at the moving averages of 0–1 hr was generated from original 5-min measurements. The fixed effect in our mixed-effects models contained covariance parameters of age, employment duration, BMI, smoking, alcohol consumption, family history of hypertension, and noise exposure. Individual subjects were treated as a random effect in the model. We also performed the mixed-effects models to compare the within-group difference of the mean values of vascular properties during work time, off-duty time, and sleep time. Three types of covariance structures were used to assess the fit of our mixed-effects regressions, including compound symmetric, unstructured, and the first-order autoregressive models. Both the compound symmetric and first-order autoregressive models met the convergence criteria that indicated the stability of the results, but the unstructured model did not meet these criteria. We chose the first-order autoregressive model as the best-fitted one because of the minimizing value of Akaike’s information criterion (Cnaan et al. 1997) in all vascular parameters except the BAC with the compound symmetric structure. The MIXED Procedure containing fixed and random effects in SAS, version 8.2 (SAS Institute Inc., Cary, NC, USA) was used to perform the linear mixed-effects regression, and the statistical significance level was set at 0.05.

## Results

In Table 1 we present a summary of the demographic characteristics and health risk factors of the 20 study subjects. These workers, from 30 to 54 years of age, had an average employment duration of 14.95 years and an average BMI of 24.57 kg/m^2^. Their mean values of resting SBP and DBP were 122.59 mmHg and 83.47 mmHg, respectively, and their average levels of total cholesterol and triglyceride were 201.60 mg/dL and 161.80 mg/dL, respectively. The prevalence rates of subjects having regular exercise, cigarette smoking, alcohol consumption, and a family history of hypertension were 25, 65, 30, and 58%, respectively. Although there were no statistically significant differences between high- and low-noise–exposed workers in these risk factors (*p* > 0.05), we found the prevalence of smoking very high in the high-noise–exposure group (73%) compared with the low-noise–exposure group (40%). We also found 12 high-noise–exposed workers with material hearing impairment defined by the U.S. Occupational Safety and Health Administration (OSHA 1981), which is an average hearing threshold level (HTL) of 25 dB at 1,000, 2,000, and 3,000 Hz.

During the study subjects’ 24-hr monitoring periods, we obtained a total of 230 available vascular parameter measurements and 1,785 personal noise readings, including 1,765 segments of 5-min Leq during working periods and 20 TWAs during off-duty periods. The full-shift TWAs (Leq) of high-noise–exposed workers (85 ± 8 dBA, mean ± SD) were significantly higher than those of low-noise–exposed workers during work periods (59 ± 4 dBA). By contrast, the full-shift TWAs (Leq) during off-duty periods showed no significant difference between the high- and low- noise–exposure groups (61 ± 7 dBA vs. 56 ± 12 dBA).

Table 2 summarizes the BAC, BAD, BAR, SVC, and SVR measured during work, off-duty, and sleeping times and over 24 hr for high- and low-noise–exposed workers. We found significant differences in work-time BAD, SVC, and SVR; off-duty time BAC, BAD, and SVC; and sleep time SVR (marginally) between the two groups. After adjusting for potential confounders, BAC values for low-noise–exposed workers were significantly higher than those for high-noise–exposed workers (by an average of 1.29 %mL/mmHg over 24 hr and 1.89 %mL/mmHg during off-duty periods). Over 24 hr and during the off-duty period, BADs were also significantly higher among low-noise–exposed workers than among high-noise–exposed workers (by averages of 1.26 %/mmHg and 1.73 %/mmHg, respectively). SVCs of low-noise–exposed workers were significantly higher than those of high-noise–exposed workers by an average of 0.19 mL/L/mmHg over 24 hr and 0.38 mL/L/mmHg (marginally, *p* = 0.07) during sleep periods. In contrast, SVRs of high-noise–exposed workers were significantly higher than those of low-noise–exposed workers by an average of 1.63 mL/L/min over 24 hr, 2.00 mL/L/min during work periods, and 1.73 mL/L/min (marginally, *p* = 0.07) during sleep periods. There was no significant difference between the average of BAR in high- and low-noise–exposed workers after adjusting for potential confounders over 24 hr, work periods, off-duty periods, and sleep periods.

Additionally, we used linear mixed-effects models to compare the within-group difference of the mean values of vascular properties at work time, off-duty time, and sleep time. We found that only the high-noise–exposed workers had decrements of BAR and SVC during work and off-duty periods compared with those during sleep time. We found no within-group differences of the mean BAC, BAD, and SVR values in either group during work and off-duty periods.

We used the concurrent measurements of noise exposure and vascular parameters during work periods among high-noise–exposed workers to estimate transient effects of noise exposure. We found nonsignificant increases of 0.10 ± 0.12 %mL/mmHg in BAC, 0.08 ± 0.08 %/mmHg in BAD, 0.01 ± 0.01 mL/ L/mmHg in SVC, and 0.07 ± 0.07 mL/L/ min in SVR but a decrease of 11.43 ± 23.79 mmHg/L/min in BAR per A-weighted decibel increase in noise exposure by the linear mixed-effects regressions. However, there were no significant changes in BAC, BAD, BAR, SVC, and SVR among high-noise–exposed workers caused by the 30-min, and 60-min time-lagged noise exposures per decibel after adjusting for age, employment duration, BMI, smoking, drinking, and family history of hypertension in the linear mixed-effects models.

The association between occupational noise exposure and 24-hr vascular parameters is summarized in Table 3. Based on a dichotomous noise exposure variable (high vs. low), our regression models showed that occupational noise exposure was significantly associated with BAC, BAD, SVC, and SVR, but not with BAR after controlling for other risk factors. Workers with TWA occupational noise exposure of 85 ± 8 dBA had lower means of 1.38 ± 0.55 %mL/mmHg BAC, 1.29 ± 0.51 %/mmHg BAD, and 0.24 ± 0.10 mL/L/mmHg SVC, but the higher mean of 1.93 ± 0.67 mL/L/min SVR over 24-hr periods compared with workers with TWA occupational noise exposure of 59 ± 4 dBA. In addition, we determined that 24-hr BAC, BAD, and SVC were significantly lower among workers with a family history of hypertension (*p* < 0.05). We also found that the 24-hr SVC was significantly higher among workers with longer employment duration (p < 0.05).

## Discussion

The present study shows that noise exposure has a sustained effect on vascular structural properties, including 24-hr-averaged BAC, BAD, SVC, and SVR of healthy male workers exposed to full-shift TWAs (Leq) of 85 ± 8 dBA. However, our findings suggest no transient effect of noise on vascular parameters at occupational exposure levels lower than full-shift TWAs (Leq) of 85 ± 8 dBA. Previous studies reported that noise stimuli > 95 dBA for 10–20 min had an increasing effect on total peripheral vascular resistance (Andrén et al. 1980; Sawada 1993a, 1993b). Such comparisons indicate that there are thresholds of exposure on noise-induced vascular structural changes.

The effects of noise on vascular structural changes reported in the present study do not come from occupational exposure alone. The between-group differences in BAC, BAD, and SVC (marginally) during the off-duty period indicate possible contributions from environmental noise exposure.

Our findings are also limited by the small number of study subjects and by unbalanced comparisons between blue-collar workers with high-noise exposure and white-collar workers with low-noise exposure. Any such differences in vascular properties could be due to selection bias or other factors, such as unknown lifestyle factors of the subjects. For example, the low-exposure workers had relatively higher SVR readings (+ 7.2%) during the off-duty time than the high-exposure group (+ 5.4%) in comparison to their baseline readings during sleep time.

Such sustained effects can also be overestimated because we did not consider all potential confounders as covariates in our analyses. Important but uncontrolled risk factors of arterial stiffness among our study subjects included blood glucose, low-density lipoprotein cholesterol, pulse pressure, vasoactive drugs, and insulin (O’Rourke and Mancia 1999; Urbina et al. 2002, 2005). Some confounders related to hypertension, such as serum uric acid, salt in diet, dietary potassium, and daily alcohol intake (Beilin et al. 1999; Viazzi et al. 2005), are not entirely excluded by results of the present study.

Our results also support the finding that people with a family history of hypertension are associated with decreased BAC, BAD, and SVC as reported in previous studies (Brinton et al. 1996; Urbina et al. 2002). Older subjects and those with higher BMIs are known to have lower arterial compliance and distensibility (Urbina et al. 2002). Although our statistical model controlled for age and BMI, such analysis may not be sufficiently robust with only 20 subjects. Therefore, the possibility of confounding effects on arterial compliance and distensibility by age and BMI cannot be completely excluded.

In the present study we applied repeated measurements to sufficiently reduce the observational variability within individuals instead of between groups (Checkoway et al. 2004). Although there were only 20 study subjects in the study, we obtained a relatively large number of noise exposure measurements and vascular parameters during 24-hr monitoring periods, including 230 available vascular parameter measurements and 1,785 personal noise readings. Accordingly, a small number of study subjects with a large amount of time-series data did not affect the determination of transient effects. However, the limited number of workers in our study may restrict the feasibility of making a detailed adjustment to confounders on all person-related factors and thus limit the extrapolation of our findings to workers in other industrial settings. For example, potential confounding may be stemming from differences in smoking habits, although this appeared to be of no significance in our mixed-effect models.

Although limited by the small sample size and some uncontrolled potential confounders, our findings generally support the conclusion that there were sustained effects in male adults at occupational noise exposure to 85 ± 8 dBA TWA at work. Our findings provide empirical evidence that the prolonged exposure to noise may cause elevated blood pressure through a sympatheticotonia-induced endothelial lesion. One possible mechanism in which prolonged occupational noise exposure may affect hypertension is sustained structural changes in vascular properties. Noise exposure may decrease the stroke volume after a short time lag (Andrén et al. 1980; Sawada 1993a) and cause early increases in BAC and BAD. The increase in SVC may occur, followed by decreases in BAR and SVR. BAR and SVR may increase through changes in BAC, BAD, and SVC to elevate blood pressure among noise-exposed workers.

Future human studies with a population-based design, more diverse subjects, and longer follow-up are still needed to confirm our findings on the sustained effects of occupational noise exposure. Future human and animal studies with more detailed measurements of toxicologic end points are still needed to illustrate the biological mechanisms of noised-induced hypertension at or below current occupational exposure levels.

## Figures and Tables

**Table 1:** Brief description of study subjects.

Characteristics	High-exposure group	Low-exposure group	Total
No. of subjects	15	5	20
Age [years (mean ± SD)]	39.27 ± 6.78	44.60 ± 8.17	40.60 ± 7.32
Employment duration [years (mean ± SD)]	14.33 ± 8.20	16.80 ± 8.87	14.95 ± 8.20
BMI [kg/m^2^ (mean ± SD)]	24.24 ± 2.16	25.57 ± 1.39	24.57 ± 2.05
Resting SBP [mmHg (mean ± SD)]	123.00 ± 19.02	120.67 ± 17.50	122.59 ± 18.25
Resting DBP [mmHg (mean ± SD)]	83.43 ± 12.38	83.67 ± 8.50	83.47 ± 11.55
Total cholesterol [mg/dL (mean ± SD)]	205.20 ± 89.26	190.80 ± 13.74	201.60 ± 77.15
Triglyceride [mg/dL (mean ± SD)]	169.60 ± 93.97	138.40 ± 49.99	161.80 ± 85.00
Regular exercise [no. (%)]			
Yes	3 (20)	2 (40)	5 (25)
No	12 (80)	3 (60)	15 (75)
Smoking [no. (%)]			
Yes	11 (73)	2 (40)	13 (65)
No	4 (27)	3 (60)	7 (35)
Alcohol consumption [no. (%)]			
Yes	5 (33)	1 (20)	6 (30)
No	10 (67)	4 (80)	14 (70)
Family history of hypertension [no. (%)]			
Yes	8 (57)	3 (60)	11 (58)
No	6 (43)	2 (40)	8 (42)
Material hearing impairment [no. (%)]^a^	12 (80)	NA	12 (80)

NA, not available.

a Defined by OSHA (1981) criteria (i.e., an average HTL of 25 dB at 1,000, 2,000, and 3,000 Hz).

**Table 2:** Values (mean ± SD) for vascular parameters over 24-hr, work time, off-duty time, and sleep time by study groups.

	Work time (0800–1630 hours)	Off-duty time (1630–2300 hours)	Sleep time (2300–0800 hours)	24-hr average
BAC (%mL/mmHg)				
High-noise–exposure group (no.)^a^	8.73 ± 2.84 (26)	7.14 ± 2.13 (63)	7.86 ± 1.97 (94)	7.74 ± 2.22 (183)
Low-noise–exposure group (no.)	9.91 ± 2.50 (13)	9.03 ± 1.37 (17)	8.34 ± 1.57 (17)	9.03 ± 1.88 (47)
Adjusted *p-*value^b^ (unadjusted)^c^	0.34 (0.21)	0.02 (0.04)	0.66 (0.42)	0.03 (0.01)
BAD (%/mmHg)				
High-noise–exposure group (no.)	6.37 ± 2.02 (26)	5.71 ± 1.75 (63)	6.70 ± 1.85 (94)	6.31 ± 1.89 (183)
Low-noise–exposure group (no.)	7.77 ± 1.87 (13)	7.44 ± 1.34 (17)	7.55 ± 1.48 (17)	7.57 ± 1.52 (47)
Adjusted *p-*value^b^ (unadjusted)^c^	0.17 (0.05)	0.02 (0.04)	0.30 (0.21)	0.03 (0.01)
BAR (mmHg/L/min)				
High-noise–exposure group (no.)	2132.73 ± 626.40 (26)^d^	3239.39 ± 1213.02 (63)^d^	3647.31 ± 1574.95 (94)	3291.69 ± 1442.49 (183)
Low-noise–exposure group (no.)	1882.81 ± 546.82 (13)	2638.26 ± 818.93 (17)	3138.95 ± 965.72 (17)	2610.40 ± 942.76 (47)
Adjusted *p-*value^b^ (unadjusted)^c^	0.38 (0.24)	0.37 (0.31)	0.71 (0.30)	0.39 (0.05)
SVC (mL/L/mmHg)				
High-noise–exposure group (no.)	1.20 ± 0.28 (26)^d^	1.22 ± 0.31 (63)^d^	1.49 ± 0.32 (94)	1.36 ± 0.33 (183)
Low-noise–exposure group (no.)	1.53 ± 0.41 (13)	1.60 ± 0.32 (17)	1.51 ± 0.23 (17)	1.55 ± 0.32 (47)
Adjusted *p-*value^b^ (unadjusted)^c^	0.33 (0.01)	0.07 (0.02)	0.15 (0.76)	0.03 (0.02)
SVR (mL/L/min)				
High-noise–exposure group (no.)	19.19 ± 2.17 (26)	19.60 ± 2.60 (63)	18.65 ± 2.48 (94)	19.06 ± 2.50 (183)
Low-noise–exposure group (no.)	17.19 ± 2.24 (13)	18.13 ± 1.78 (17)	16.92 ± 1.95 (17)	17.43 ± 2.01 (47)
Adjusted *p-*value^b^ (unadjusted)^c^	0.02 (0.02)	0.86 (0.11)	0.07 (0.06)	0.02 (0.01)

a Number of measurements for specific vascular parameters.

b The between-group differences were tested using linear mixed-effects regression models adjusted for age, employment duration, BMI, smoking, drinking alcohol, regular exercise, and family history of hypertension.

c Linear mixed-effects regressions were used to test the between-group without adjustments of potential confounders.

d The significant within-group differences of the mean values compared with those at sleep time (reference) by study groups (*p* < 0.05).

**Table 3:** The association between occupational noise exposure (high- vs. low-noise–exposure groups) and 24-hr vascular parameters using linear mixed-effects model.

Model^a^	BAC (%mL/mmHg) β (SE)	BAD (%/mmHg) β (SE)	BAR (mmHg/L/min) β (SE)	SVC (mL/L/mmHg) β (SE)	SVR (mL/L/min) β (SE)
High- vs. low-noise–exposure	−1.38 (0.55)**	−1.29 (0.51)**	426.20 (469.22)	−0.24 (0.10)**	1.93 (0.67)**
Family history of hypertension (yes/no)	−1.19 (0.48)**	−1.00 (0.45)**	566.28 (419.20)	−0.28 (0.09)**	1.12 (0.59)*
Employment duration (years)	−0.10 (0.06)	−0.12 (0.06)*	4.51 (55.32)	−0.03 (0.01)**	0.06 (0.08)
Age (years)	0.04 (0.08)	0.08 (0.07)	2.94 (69.38)	0.02 (0.01)	−0.04 (0.10)
BMI (kg/m^2^)	0.19 (0.11)	0.11 (0.11)	−120.30 (97.68)	0.01 (0.02)	−0.03 (0.14)
Smoking (yes/no)	−0.20 (0.64)	0.13 (0.60)	166.18 (559.58)	−0.04 (0.11)	−1.19 (0.79)
Alcohol consumption (yes/no)	0.07 (0.48)	0.04 (0.45)	465.04 (417.08)	0.19 (0.09)*	1.23 (0.59)*
Regular exercise (yes/no)	−0.21 (0.44)	−0.39 (0.42)	365.72 (385.24)	−0.07 (0.08)	0.10 (0.54)

a The linear mixed-effects regression model is adjusted for age, employment duration, BMI, smoking, drinking alcohol, regular exercise, and family history of hypertension.

* *p* < 0.1.

** *p* < 0.05.
